# A finite element analysis of the effects of archwire size on orthodontic tooth movement in extraction space closure with miniscrew sliding mechanics

**DOI:** 10.1186/s40510-018-0255-8

**Published:** 2019-01-21

**Authors:** Jun Kawamura, Naohiko Tamaya

**Affiliations:** 1 Kawamura Dental Office, 4-21 Sodensakae-machi, Gifu, 502-0847 Japan; 2 Tamaya Orthodontic Office, Fukui, Japan

**Keywords:** Sliding mechanics, Miniscrew, Extraction space closure, Archwire, Finite element method

## Abstract

**Background:**

Sliding mechanics with miniscrews is recently used for extraction space closure. The purpose of this study was to elucidate how and why the archwire size affects long-term tooth movement in miniscrew sliding mechanics.

**Methods:**

Long-term orthodontic tooth movements were simulated based on a remodeling law of the alveolar bone by using a finite element method, in which the bracket rotated freely within a clearance gap (a play) of the archwire-bracket slot. The archwire size was changed to 0.021, 0.018, and 0.016 in. for the 0.022-in. bracket.

**Result:**

Lingual crown tipping and extrusion of the incisors increased with decreasing the archwire size. Movements of the posterior teeth were approximately the same irrespective of archwire size.

**Conclusions:**

When decreasing the archwire size, a play of the archwire-bracket slot, as well as the elastic deformation of the archwire, resulted in lingual tipping of the incisors. This tipping led to extrusion of the incisors.

## Introduction

Sliding mechanics is a standard method used for extraction space closure [1, 2]. Although the retraction force decays by friction between the archwire and the bracket slot, the bracket slides along the archwire so that bodily tooth movement can be easily achieved. This is an advantage over segmental or sectional mechanics, in which elaborate appliance designs are necessary to achieve bodily tooth movement.

Miniscrews placed into the maxillary or mandibular bone have recently been used for an orthodontic anchorage in the sliding mechanics [3–5]. This method, miniscrew sliding mechanics, eliminates anchorage problems and easily achieves en masse retraction of the anterior teeth. A most important problem in this method is the rotation of the occlusal plane of the entire dentition, which depends on the direction of the force applied from the miniscrew. This effect of the force direction has been clarified by a clinical study and also confirmed by a finite element study carried out by the authors [6, 7]. To move the teeth into the desired position, clinicians must select an appropriate force direction, which is achieved by a proper combination of miniscrew positions and power arms.

In the previous study, the authors assumed that the archwire size (0.018 in.) was the same as the bracket slot so that the archwire was securely fixed to the bracket [7]. However, archwires with a smaller size than a bracket slot have been used in many clinical situations [3–5]. In these cases, a clearance gap (a play) between the archwire and the bracket slot exists, and thus, the bracket rotates freely within the space. In addition, when decreasing the archwire size, bending stiffness of the archwire decreases. These effects have not been investigated from a mechanical point of view. The purpose of the present study is to elucidate how and why the archwire size affects long-term tooth movement in miniscrew sliding mechanics. For this purpose, the orthodontic movement was simulated by a finite element method that has been developed by the authors [8–10].

## Materials and methods

Because the present simulation method is closely related to the previous studies, only the main principles are explained below [8–10].

### Analysis model

After extraction of the maxillary first premolars, the six anterior teeth were retracted distally using miniscrew sliding mechanics. Assuming symmetry for both sides of the arch, a model of only the left side was fabricated.

Finite element models of the teeth were made based on dental study model (i21D-400C, Nissin Dental Products Inc., Kyoto, Japan). The method consists of three steps: first, sectional images of the dental study model were taken using dental cone beam computed tomography (CBCT), AZ300CT (Asahi Roentgen, Co., Ltd., Kyoto, Japan); second, using 3D modeling software, 3D-Doctor (Able Software Corp., Lexington, Massachusetts, USA), stereolithographic (STL) model of each tooth was constructed; third, the STL models were converted to finite element models using meshing software, ANSYS AI*Environment (ANSYS, Inc., Canonsburg, Pennsylvania, USA).

The above finite element models were used to calculate the tooth elements whose elastic responses were identical to the teeth supported with the periodontal ligament (PDL) [8]. The tooth element has two nodes corresponding to the bracket on the tooth and the alveolar socket of the tooth. In calculating this element, the tooth and the alveolar bone were rigid bodies while the PDL was a linear elastic film with uniform thickness of 0.2 mm. These assumptions enabled the simulation method to be very simple. Young’s modulus and Poisson’s ratio of the PDL were assumed 0.13 MPa and 0.45, respectively, so that mobility of the second premolar calculated by using these values became about the same as those measured in vivo [10]. In addition, the authors have confirmed non-linear elastic property of the PDL had almost no effect on long-term orthodontic tooth movement [11].

**Fig. 1:**
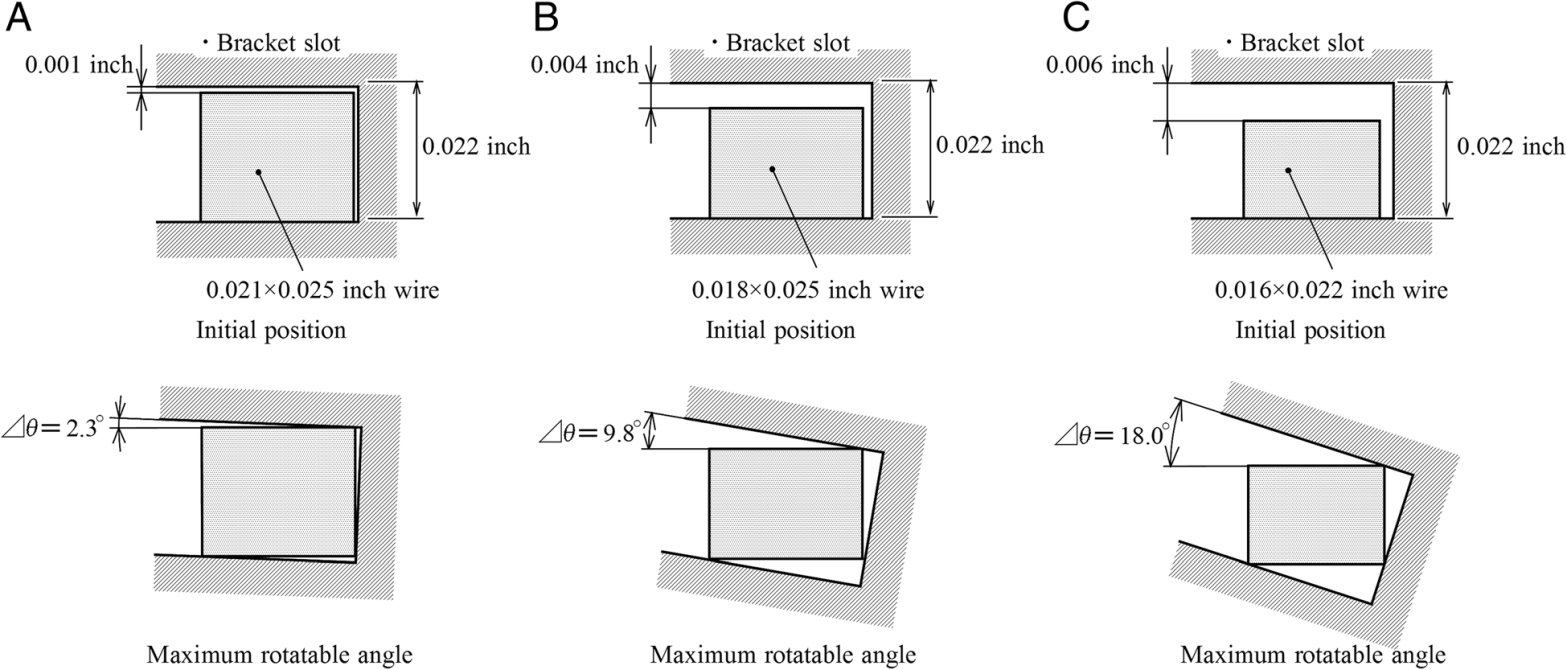
Initial positions and maximum rotation angles, Δθ, when archwires of 0.021, 0.018, and 0.016 in. are inserted into a bracket of 0.022 in.. **a** 0.021 in. **b** 0.018 in. **c** 0.016 in

Three different archwire sizes, 0.021, 0.018, and 0.016-in. archwires, were inserted into a 0.022-in. bracket slot, respectively. Initial positions of archwire in the bracket slot were assumed as shown in Fig. 1a–c. In these cases, the bracket rotated freely within a play of the archwire-bracket slot. The maximum rotation angles, Δθ, were 2.3, 9.8, and 18.0°, respectively.

A power arm of 4 mm in length was bonded to the archwire between the lateral incisor and the canine. The archwire and the power arm, which were made from a 0.018 × 0.025-in. stainless steel wire (Young’s modulus: 200 GPa), were divided into the three-dimensional elastic beam elements. The tooth elements were connected to the archwire through three-dimensional spring elements that simulated contact condition between the archwire and the bracket slots. The frictional coefficient between them was assumed *μ* = 0.15 referring other experimental data [12].

A miniscrew was placed into the buccal side of a maxillary bone between the second premolar and the first molar in a position 8 mm gingival to the archwire. Retraction force of 1.5 N (150 g force) was applied to the power arm from the miniscrew.

Under the above assumptions, the finite element model for simulating the long-term orthodontic movement was constructed of the archwire, the power arm, the spring elements, and the tooth elements.

### Simulation of orthodontic tooth movement

Orthodontic movement was simulated by the three steps: first, forces and moments acting on the teeth were calculated using the finite element model; second, the amount and direction of movement were calculated for each tooth based on the stresses induced in the PDL; third, by using their amount and direction, the alveolar socket of each tooth moved. By repeating these three steps, the teeth moved step by step. The force system acting on the teeth was updated during each step. The authors have developed a finite element computer program for simulating long-term orthodontic tooth movement [8–10]. Pre-post processor of finite element method, FEMAP V6.0 (Enterprise Software Products, Inc., Exton, PA, USA), was used for illustrating the tooth movement and the deformation of the archwire.

The amount and direction of orthodontic tooth movement were calculated based on resorption and apposition of the alveolar bone. This bone remodeling rate was assumed to be in proportion to the mean stress in the PDL. Progress of the tooth movement is dependent on a parameter *CT*, where *C* is the amount of bone remodeling (μm) per unit time (day) and unit stress (kPa), and *T* is elapsed time in days. Thus, a unit of *CT* becomes μm/kPa. Although the C was estimated at 6 μm/(kPa·day) in the previous study, this value has not been validated [7].

## Results

**Fig. 2:**
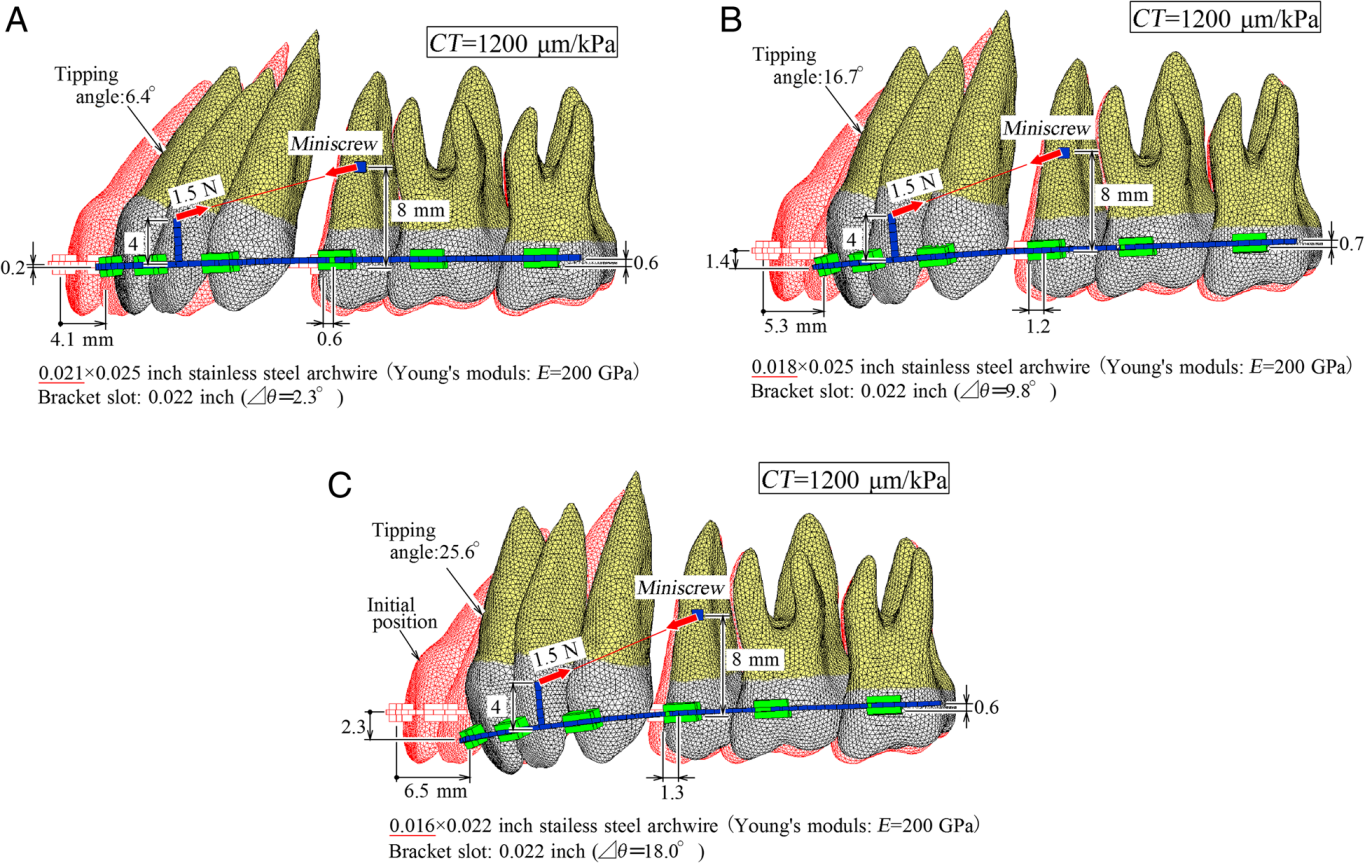
Movement patterns when archwires of 0.021, 0.018, and 0.016 in. are inserted into brackets of 0.022 in.. Lingual tipping and extrusion of the incisor increased when decreasing the archwire size. Movements of the posterior teeth were about the same irrespective of the archwire size. **a** 0.021 in. **b** 0.018 in. **c** 0.016 in

Figure 2a–c illustrate tooth movements at *CT* = 1200 μm/kPa in three cases when 0.021, 0.018, and 0.016-in. archwires were inserted into 0.022-in. brackets, respectively. In these figures, initial tooth positions are illustrated with red outlines.

The central incisor moved distally by approximately 5 mm. When decreasing the archwire size, lingual tipping and extrusion of the incisors increased. In comparison with the anterior teeth, movements of the posterior teeth were approximately the same irrespective of archwire size.

## Discussion

### The effect of archwire size on tooth movement

In the case of a 0.021-in. archwire (Fig. 2a), although the central incisor tipped lingually by 6.4°, the root apex moved distally so that the anterior teeth could move bodily. However, in the case of 0.018 and 0.016-in. archwires, root apices of the incisor moved mesially, namely the occurrence of lingual tipping. The lingual tipping rapidly moved the incisor’s crown, and the distal movement of the incisor increased when decreasing the archwire size. It was generally agreed that a play of the archwire-bracket slot should be reduced in order to control precisely the tooth movement.

Immediately after the retraction force was applied, the bracket rotated within a play of the archwire-bracket slot, and the incisor tipped lingually thereby. This tipping, which was produced without moment, was a kind of uncontrolled tipping. The lingual tipping continued till the tipping angle reached the maximum rotation angle Δθ so that both diagonal corners of rectangular section of the archwire contacted to the bracket slot (Fig. 1). After that, the lingual tipping was prevented by the archwire. This is one of the reasons why the lingual tipping angle of the incisor increased with decreasing the archwire size. A play between the archwire and bracket slot produces lingual tipping in all the teeth whenever their brackets move. This tipping cannot be prevented by using an inversely prebent archwire. In clinical situations, the amount of tipping due to a play changes depending on the initial position of archwire in the bracket slot. When an archwire is placed into the bracket slot with a clearance gap as shown in the top illustrations of Fig. 1, the maximum tipping occurs. When an archwire is initially in contact with the bracket slot as shown in the bottom illustrations of Fig. 1, tipping due to a play does not occur. This initial contact will be caused by using an archwire with an initial twist or bend and by a misalignment between the archwire and bracket slot.

By observing the tooth movement especially in Fig. 2c, we can understand that bowing caused by elastic deflection of the archwire was another reason for lingual tipping of the incisors. This tipping occurred under a condition where a moment was applied from the archwire to the incisor, and therefore, it was a kind of controlled tipping. The tipping angle of the incisor was greater than the maximum rotation angle Δθ in each archwire size. The difference between both angles is due to the elastic deflection of the archwire, which increases by decreasing the archwire size. In Fig. 2, the tipping angle due to the elastic deflection becomes 4.1° (= 6.4–2.3°), 6.9° (= 16.7–9.8°), and 7.6° (= 25.6–18.0°) when decreasing the archwire size. Elastic deflection of the archwire affected individual teeth differently. In the central incisor, an elastic deflection caused lingual tipping, and it was added to that produced by the play. As a result, lingual tipping of the central incisor became remarkable. In the canine, crown distal tipping was caused by an elastic deflection of the archwire. In the molars, the influence of elastic deflection was small. In all kinds of tooth, tipping due to the elastic deflection can be prevented by using an inversely prebent archwire or torqueing (third-order bend) of archwire [13].

Elastic deformation is inversely proportional to Young’s modulus of the archwire [14]. If a Ni-Ti super elastic archwire or a TMA (Ti-Mo alloy) archwire was used in place of a stainless steel archwire, the amount of the elastic deflection will more than treble. This is the reason why low stiffness archwires are unsuitable for any sliding mechanics. In addition, the amount of the elastic deflection is proportional to the retraction force *P* [14]. If the *P* increases from 1.5 N to 3.0 N, the tipping angle of the incisor due to the elastic deflection will also increase twice.

Lingual tipping, or rotation of the incisor in the sagittal plane, leads to the extrusion, which may result in overbite. In Fig. 2, the extrusion increased together with the lingual tipping when decreasing the archwire size. This secondary movement is apt to be overlooked. Clinicians should pay attention to not only lingual tipping but also extrusion of the incisor, when using a light archwire in the miniscrew sliding mechanics. This caution is also valid for the conventional sliding mechanics without a miniscrew.

In the three archwire sizes, although the posterior teeth slightly rotated counterclockwise, the distal movements were very small. This is the most advantage over the conventional sliding mechanics in which the posterior anchorage teeth move mesially to some extent. In the case of a 0.021-in. archwire (Fig. 2a), an occlusal plane of the entire dentition hardly rotated during the space closing. Movement of the entire dentition is very sensitive to the force direction [6, 7]. If a shorter power arm or a lower miniscrew position is used, the counterclockwise rotation of the entire dentition will increase. The effect of force direction in the miniscrew sliding mechanics has been discussed in detail in the previous study [7].

In Fig. 2a, a combination of a 0.021 × 0.025-in. wire and a 0.022-in. bracket was used to simulate an ideal case where there was almost no play between the archwire and the bracket slot. This combination is not usually used in clinical settings, because the play is so small that an archwire may not slide smoothly along the bracket slot.

It was found that tipping of the anterior teeth was due to three causes, a play of the archwire-bracket slot, an elastic deflection of the archwire, and a rotation of the entire dentition. Among these three causes, only the tipping due to an elastic deflection of archwire can be prevented by using an inversely prebent archwire or torqueing (third-order bend). When using such archwires, only tipping due to elastic deflection decreased, but the other types of tipping are left. If using a prebent archwire or a third-order bend to diminish tipping of the incisor caused by a play, the other teeth will be tipped as a reaction against the upright direction of the incisor. It is due to the law of action and reaction.

Based on the simulation results, the authors propose the following recommendations to achieve a tooth movement without undesired tipping in clinical settings. To prevent tipping due to a play between the archwire and the bracket slots, the archwire should be twisted initially so that contact is just made between the archwire and the bracket slots. It is necessary to predict tipping direction and twist the archwire in its opposite direction. Tipping due to an elastic deflection can be prevented by using an initially prebent archwire or torqueing (third-order bend). But an optimal amount of the prebend or the torqueing is unknown before treatment. Movement pattern of the entire dentition can be controlled by the force direction in respect to the center of resistance (CR) of the entire dentition [7]. But a correct position of the CR in a patient’s dentition is uncertain. For these reasons, an optimal condition for desired movement cannot be selected before treatment. In such situations, when undesired tipping is observed in clinical treatments, its causes should be identified at first. Then, reasonable methods should be taken to prevent the tipping in accordance with the causes.

### Advantage and limitation of the present simulation

The finite element method that has been developed by the authors can simulate three-dimensional long-term tooth movements [8–10]. This method will be like no other in orthodontics. Observing the simulated results in Fig. 2, we can easily understand how the archwire size affects the tooth movement.

It was found that two mechanisms produced lingual tipping of the anterior teeth when decreasing the archwire size. One mechanism was rotation of the bracket within a play between the archwire and the bracket slot. It will be easily understood without the present simulation. The other mechanism was elastic deflection of the archwire. In addition to that, the lingual tipping led to extrusion of the anterior teeth. These archwire’s deflection and incisor’s extrusion may not be imagined without the present simulation. This is a reason for necessitating such finite element simulations.

In the present simulation, effects of the archwire size could be examined under the same property of tooth movement. This is an advantage of the finite element simulation over randomized controlled trials (RCT), where the effect of the archwire size may be buried under the individual difference in tooth movement. Although such a finite element simulation can never take the place of RCT, it is of help to understand how and why a mechanical factor affects tooth movement.

In the present method, orthodontic tooth movement was assumed to occur by resorption and apposition of the alveolar bone (bone remodeling) depending on the mean stress in the PDL. This assumption could not be verified because the biological mechanism of the orthodontic tooth movement has not been fully clarified. Most importantly, the orthodontic tooth movement is controlled by the elastic response of the PDL through the remodeling law. The long-term orthodontic tooth movement therefore occurs in accordance with the initial tooth movement produced by elastic deformation of the PDL. This is an essential principle of the present simulation. This principle, which means that the initial tooth movement is a predictor of the orthodontic tooth movement, will be generally accepted to many clinicians and has been also confirmed by an animal experiment [15]. This is the reason why the authors believe tooth movements simulated by the present method are similar to those in clinical situations. In the present case where many teeth were connected with an archwire, the initial tooth movements of each tooth had to be updated at the current force system. Other assumptions used in the present method have been already discussed in the previous studies [8–11].

The present study must be objectively evaluated. But it is difficult to find comparable data to the present simulation results. Several finite element studies have been carried out about the miniscrew sliding mechanics [16–18]. Although their purposes were different from the present study, tooth movements only at the initial force system have been calculated by using precise finite element models. Their results were very useful for understanding of the initial movement; however, their movement patterns will be different from those of the long-term movement.

## Conclusions

In en masse space closure with miniscrew sliding mechanics, the finite element simulations illuminated the effect of the archwire size on the tooth movement.

When decreasing the archwire size, a play of the archwire-bracket slot, and also an elastic deformation of the archwire, resulted in lingual tipping of the incisors. This tipping led to an extrusion of the incisor. Clinicians should pay attention to not only lingual tipping but also extrusion of the incisors, when using a light archwire in the sliding mechanics.

## Abbreviations

CBCT: Cone beam computed tomography
PDL: Periodontal ligament
RCT: Randomized controlled trials
STL: Stereolithographic
TMA: Ti-Mo alloy
